# Dr. Elkinton’s Case of Apoplexy

**Published:** 1842-08-13

**Authors:** John A. Elkinton

**Affiliations:** Philadelphia


To the Editors of the Medical Examiner.

Gentlemen,—In looking over some papers to-day, I found my notes of the autopsy of Mrs. Dilks, about which I spoke to you some weeks ago. The case was certainly a very remarkable one of "severe apoplexy," (hæmorrhagie foudroyante.) The notes are at your service, with an account of the autopsy, from my friend, Dr. Ashmead, who made the examination.

Your obedient servant,

John A. Elkinton, M. D.

*Philadelphia*, August 2, 1842.

### *Case of Mrs. Elizabeth Dilks.*

March 25, 1842. The patient, aged 52 years, was found lying in the second story, about half past two o'clock P. M., the 24th, yesterday. She was able to speak, and said she had had a fall in the cellar. Her first attendants, under the impression she had fainted, opened the windows, and gave her spirits of camphor and water. I found her about thirty minutes after she was first discovered, (she was seen at the front door, well, at half past twelve to one o'clock the same day,) with livid surface, apparently the effects of cold. Pulse tense, wiry; very slight, if any, perceptible stertorous breathing; consciousness retained; placed her hand frequently to right side of her head, and occasionally vomited; indistinct articulation, but evidently aware of her situation, and what was going on around her. Mustard plasters were applied to the ankles, and she was covered with blankets; the hands and feet placed in hot water, and revulsives generally instituted. Cups were ordered to the temples, behind the ears, and wherever they could be applied to the head. In about two hours the pulse intermitted, and apoplectic symptoms were clearly developed—pouting of the lips, stertor, &c. I opened the temporal artery, which bled very freely. Mustard was applied between the shoulders, the hair removed from the head, &c., and while the blood was flowing freely from the divided artery she died.

*Autopsy.*

The congestion of the vessels of the scalp and cranium was enormous, and several ounces of thick dark blood escaped on raising the scalp. Considerable extravasation existed on the pericranium beneath the cups.

Arachnoid and pia mater transparent; no thickening nor inflammation; no effusion of serum on upper surface of brain.

On removing the cranium, there was also great congestion of all the vessels of the dura mater, and they poured forth blood profusely.

Vessels of pia mater all turgid with black blood.

A large quantity of bloody water at base of brain.

A coagula of blood in the separation of the arachnoid and pia mater at the base of the brain, in front of the pons.

Cortical and medullary portions more than usually injected. Their substance of a natural firmness.

Left lateral ventricle and its anterior and posterior cornu filled and much distended with serum and fluid blood.

The lacerated vessel was that between the junction of the corpus striatum and thalam. nerv. opt. of right side. This lateral sinus, with its *anterior* and *posterior* cornui, was filled and greatly distended with coagulated blood, which seemed to have been forced mechanically into it; lacerating the substance of this thalamus, and the substance of the brain over the inferior cornu, so as to compress it completely, and prevent all hæmorrhage into it.

The substance of the brain next the coagula was a mere pulp, intermixed with small coagula.

A coagulum was found in the centre of the substance of the pons varolii.

The amount of blood found in the vessels of brain, and the effusion, was calculated at sixteen ounces.

The coats of the vessels at base of brain, and in the fissure of Sylvius, were thickened with numerous small patches of a yellow, opaque, cartilaginous transformation.

